# A Safe GDNF and GDNF/BDNF Controlled Delivery System Improves Migration in Human Retinal Pigment Epithelial Cells and Survival in Retinal Ganglion Cells: Potential Usefulness in Degenerative Retinal Pathologies

**DOI:** 10.3390/ph14010050

**Published:** 2021-01-11

**Authors:** Alicia Arranz-Romera, Maria Hernandez, Patricia Checa-Casalengua, Alfredo Garcia-Layana, Irene T. Molina-Martinez, Sergio Recalde, Michael J. Young, Budd A. Tucker, Rocío Herrero-Vanrell, Patricia Fernandez-Robredo, Irene Bravo-Osuna

**Affiliations:** 1 Pharmaceutical Innovation in Ophthalmology (InnOftal), Research Group (UCM 920415), Department of Pharmaceutics and Food Technology, Faculty of Pharmacy, Complutense University, Plaza de Ramón y Cajal, s/n, 28040 Madrid, Spain; a.arranz@ucm.es (A.A.-R.); pcheca11@hotmail.com (P.C.-C.); iremm@ucm.es (I.T.M.-M.); rociohv@ucm.es (R.H.-V.); 2 Retinal Pathologies and New Therapies Group, Experimental Ophthalmology Laboratory, Department of Ophthalmology, Clínica Universidad de Navarra, 31008 Pamplona, Spain; aglayana@unav.es (A.G.-L.); srecalde@unav.es (S.R.); pfrobredo@unav.es (P.F.-R.); 3 Navarra Institute for Health Research, IdiSNA, 31008 Pamplona, Spain; 4 Red Temática de Investigación Cooperativa Sanitaria en Enfermedades Oculares (Oftared), 31008 Pamplona, Spain; 5 Instituto Universitario de Farmacia Industrial (IUFI), Faculty of Pharmacy, Complutense University, 28040 Madrid, Spain; 6 Instituto de Investigación Sanitaria del Hospital Clínico San Carlos (IdISSC), Plaza de Ramón y Cajal, s/n, 28040 Madrid, Spain; 7 Department of Ophthalmology, Schepens Eye Research Institute, Harvard Medical School, Harvard University, 20 Staniford Street, Boston, MA 02114, USA; mikey@vision.eri.harvard.edu; 8 Institute for Vision Research, Department of Ophthalmology and Visual Sciences, University of Iowa, Iowa City, IA 52242, USA; budd-tucker@uiowa.edu

**Keywords:** retinal diseases, GDNF, BDNF, RPE migration, co-delivery, PLGA microspheres, retinal ganglion cells

## Abstract

We assessed the sustained delivery effect of poly (lactic-co-glycolic) acid (PLGA)/vitamin E (VitE) microspheres (MSs) loaded with glial cell-derived neurotrophic factor (GDNF) alone (GDNF-MSs) or combined with brain-derived neurotrophic factor (BDNF; GDNF/BDNF-MSs) on migration of the human adult retinal pigment epithelial cell-line-19 (ARPE-19) cells, primate choroidal endothelial (RF/6A) cells, and the survival of isolated mouse retinal ganglion cells (RGCs). The morphology of the MSs, particle size, and encapsulation efficiencies of the active substances were evaluated. In vitro release, 3-(4,5-dimethylthiazol-2-yl)-2,5-diphenyltetrazolium bromide (MTT) cell viability, terminal deoxynucleotidyl transferase (TdT) deoxyuridine dUTP nick-end labelling (TUNEL) apoptosis, functional wound healing migration (ARPE-19; migration), and (RF/6A; angiogenesis) assays were conducted. The safety of MS intravitreal injection was assessed using hematoxylin and eosin, neuronal nuclei (NeuN) immunolabeling, and TUNEL assays, and RGC in vitro survival was analyzed. MSs delivered GDNF and co-delivered GDNF/BDNF in a sustained manner over 77 days. The BDNF/GDNF combination increased RPE cell migration, whereas no effect was observed on RF/6A. MSs did not alter cell viability, apoptosis was absent in vitro, and RGCs survived in vitro for seven weeks. In mice, retinal toxicity and apoptosis was absent in histologic sections. This delivery strategy could be useful as a potential co-therapy in retinal degenerations and glaucoma, in line with future personalized long-term intravitreal treatment as different amounts (doses) of microparticles can be administered according to patients’ needs.

## 1. Introduction

Age-related macular degeneration (AMD) is the leading cause of blindness in individuals over age 55 years old [1,2]. Advanced AMD is characterized by geographic atrophy (GA), choroidal neovascularization (CNV), or both [3,4], and exhibits similar initiating molecular, cellular alterations, and oxidative damage [5,6]. GA affects approximately 90% of AMD patients and is characterized by severe visual impairment due to damage to the retinal pigmented epithelial (RPE) cells, Bruch’s membrane (BM), and choriocapillaris and loss of photoreceptors [7,8]. Glaucoma is the second leading cause of blindness with 60.5 million patients diagnosed worldwide in 2010, a figure that was expected to increase to 79.6 million by 2020 [9]. Glaucoma is characterized by a loss of retinal ganglion cells (RGCs), and several preclinical studies have demonstrated that neurotrophins (NTs) prevent RGC loss [10].

In neovascular AMD (nvAMD, 10% of AMD cases) new capillaries sprout from the choroid through the BM and enter the retina, resulting in CNV. Therefore, inhibition of angiogenesis is critical in the prevention and treatment of nvAMD [11] and intravitreal anti-vascular endothelial growth factor (anti-VEGF) is the gold standard therapy [12,13,14]. Repeated doses of anti-VEGF are needed for several years, because cessation may result in the recurrence of CNV [15]. Although anti-VEGF has been shown to be safe, side effects, mainly related to the injection procedure, have occurred [16]. In addition, VEGF is an essential factor for cell survival [17] and repeated exposure to these drugs may damage retinal neurons [18,19]. However, other studies suggest that those effects might occur as a consequence of the natural disease progression [20]. Irrespective of the disease aetiology and treatment administered, at the final stage of the disease, the RPE–photoreceptor interface is damaged.

Consequently, new targets and novel VEGF-independent pathway approaches are under investigation. However, large clinical phase III trials of combined anti-VEGF/platelet-derived growth factor inhibition (Pegpleranib) in nvAMD, targeting pericytes, have failed to improve visual acuity at 12 months compared to anti-VEGF monotherapy [21,22].

The neuroprotective properties of neurotrophic factors (NTFs) make them a useful therapeutic approach for the treatment of retinal degenerative diseases [23,24]. Brain-derived neurotrophic factor (BDNF) concentrations in aqueous humour decreased in the AMD patients with outer retinal layer thicknesses [25]. Intravitreal administration of exogenous glial-derived neurotrophic factor (GDNF) to these patients has demonstrated strong neurotrophic properties in the retina. This NTF was able to reduce retinal ganglion cells (RGC) and their axonal death in an animal model of optic nerve damage [23]. It also preserved photoreceptor cell functionality in an rd mouse model, enhancing rod outer segment maintenance and increasing the number of photoreceptors expressing opsin [26,27]. In addition, BDNF was observed to be downregulated in an oxidative stress-related environment (high glucose) in adult RPE cell line-19 (ARPE-19) cells [28]. Proliferating peripheral RPE cells have demonstrated the ability to migrate into central senescent regions of the retina to recover age-related central RPE loss [29]. Interestingly, the mature RPE is held in a state of quiescence by the adjacent neural retina, and RPE cells proliferate in response to retinal degeneration [30]. The repair mechanisms within injured retinas involve a significant glial cell reaction marked by glial cell proliferation and migration from their original location toward the injury site, followed by overproduction of NTFs such as BDNF, GDNF and neurotrophin-3 [31]. Furthermore, BDNF has been demonstrated to promote cell differentiation and inhibit inflammation under pathological conditions in the RPE [32,33]. Therefore, the artificial supplementation of these compounds might offer an interesting therapeutic benefit. Moreover, clinical observations have shown that the RPE has very limited ability to regenerate leading to photoreceptor death and irreversible blindness [34]; therefore, increasing intraocular NTF levels might solve this problem [31]. This is the rationale of Neurotech (Cumberland, RI, USA), a capsule of polyethylene terephthalate developed to be implanted into the vitreous cavity and anchored at the pars plana. This device contains genetically modified human RPE cells to overexpress ciliary NTF (CNTF). Phase I and II trials in patients with GA in AMD and retinitis pigmentosa have demonstrated that the device is well tolerated and has shown some evidence of efficacy [35,36].

The treatment of chronic retinal diseases such as AMD and glaucoma requires an effective local concentration of the active agents for prolonged periods of time [37,38]. High-dose and repeated intravitreal injections are necessary to maintain drug levels in the therapeutic range [38,39]. In this regard, new concepts have emerged with the use of biodegradable microparticulate systems to provide an alternative to multiple injections. These strategies control the release of the active substances over time and reduce the frequency of intraocular injections and their associated risks (such as retinal detachment, endophthalmitis, glaucoma, vitreous hemorrhage, and cataract) [40,41]. Poly (lactic-co-glycolic) acid (PLGA) microspheres (MSs) are largely inert in the vitreous cavity, biocompatible, tolerable [42,43], and can be easily eliminated from the body [44,45]. Therefore, the use of MSs formulated with PLGA plus vitamin E (VitE) and loaded with NTFs offers promise for retinal chronic diseases treatment [46]. In this context, GDNF-loaded PLGA/VitE MSs have functionally and structurally rescued photoreceptors in a rhodopsin knockout mouse model (rho^−/−^) [47].

The treatment of ocular multifactorial diseases using a combination of more than one active substance is currently in the research pipeline [48]. In fact, the combination of two or more active substances has already demonstrated positive effects; different pathways of the diseases are blocked at multiple levels. For example, the co-administration of GDNF and docosahexaenoic acid showed an additive effect both on photoreceptor survival and on opsin expression [49]. In addition, intravitreal co-administration of GDNF and BDNF in solution to an animal model of optic nerve axotomy prolonged the survival of RGC more than the individual injection of either agent alone did [50,51]. However, co-administration of the free active compounds shows limited effects because of events such as short-half lives, adverse effects, or unpredictable in vivo distributions [52]. Therefore, the applicability of a synergetic therapy in chronic retinal diseases is totally linked to the development of devices that are able to sustain therapeutic drug concentrations in target tissues for extended periods of time [53]. Consequently, we have proposed a novel sustained-release intraocular drug delivery system based on multi-loaded PLGA-MSs incorporating three recognized neuroprotective agents (dexamethasone, melatonin, and coenzyme Q10) as an interesting platform that has been useful in RGC rescue in an animal model of ocular hypertension [54].

The aim of the present work was to develop a PLGA/VitE microparticulate platform that can co-encapsulate and co-deliver two NTFs (GDNF and BDNF) as a potential therapy against retinal diseases with associated RPE and RGC damage. To this end, the different microparticulate systems prepared were physicochemically characterized and then corresponding toxicity studies were performed. Moreover, in vitro and in vivo cell migration and angiogenesis were assessed in RPE and choroidal endothelial cells, respectively, and as a bioactivity study was conducted in cultured RGCs after GDNF/BDNF release.

## 2. Results

### 2.1. MSs Characterization

The encapsulation method proposed in this work led to a high production yield in both NTF-loaded formulations, similar to those obtained for control MSs.

**Figure 1:**
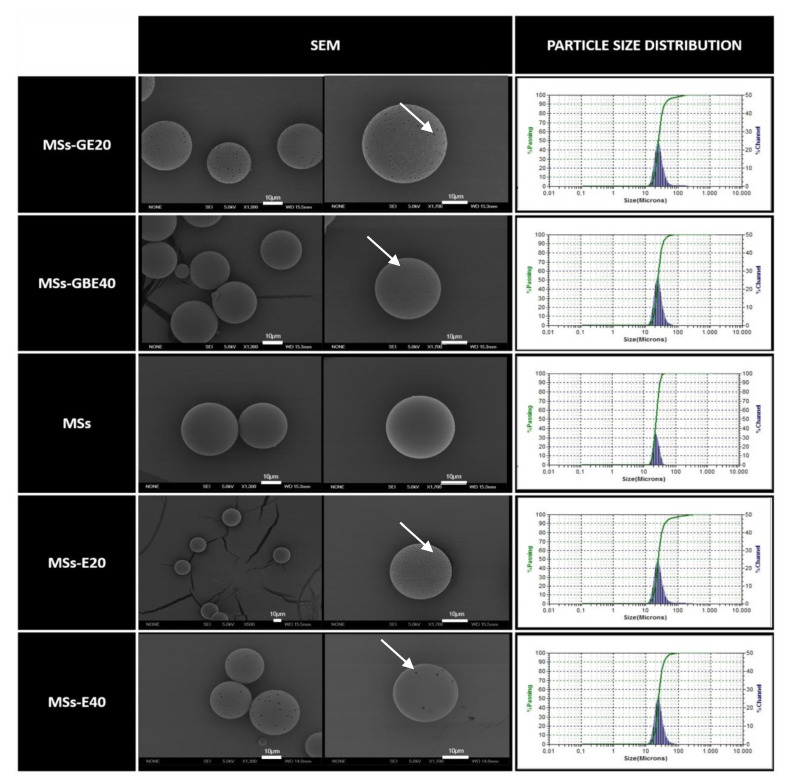
Microsphere (MS) characterization. Morphological evaluation by scanning electron microscopy (SEM) and particle size distribution. Blank MSs (MSs); MSs/VitaminE(20) (MSs-E20); MSs/VitaminE(40) (MSs-E40) GDNF/VitE(20)-loaded PLGA MSs (MSs-GE20); GDNF/BDNF/VitE(40)-loaded PLGA microspheres (MSs-GBE40). SEM investigation showed the presence of spherical particles with comparable and regular size distributions, which were confirmed by particle size measurements. White arrows: pores on the MS surfaces. Scale bar: 10 µm.

Figure 1 shows the external morphology of the different polymeric microparticles obtained. Scanning electron microscopy (SEM) pictures revealed well-defined spherical MSs in all cases (Figure 1, SEM images). Surface morphological differences were observed between blank PLGA MSs and loaded MSs. While the microspheres formulation prepared with plain PLGA showed a smooth appearance, loaded MSs exhibited a number of small pores with a rough surface. The microencapsulation method was optimized to obtain particles in the 20–38 µm range, suitable for injection as a suspension through needles typically used for intravitreal administration (25 G–32 G) [45]. The particle size distribution presented in Table 1 denoted mean particle size values similar for all batches.

### 2.2. In Vitro Release Studies

**Figure 2:**
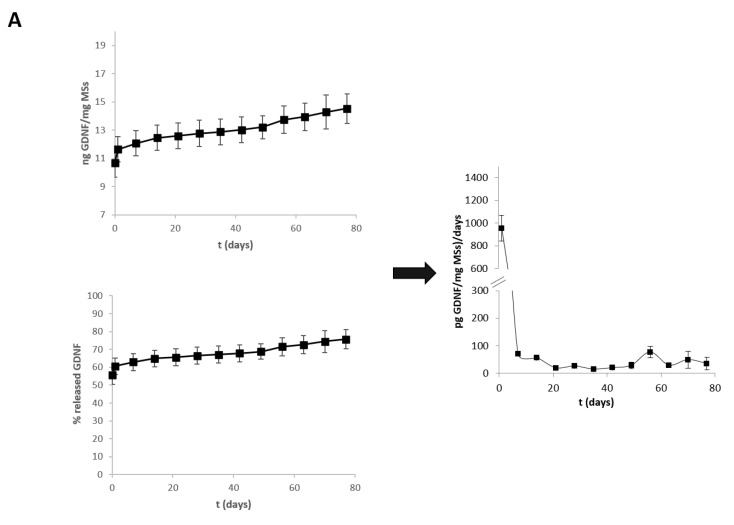

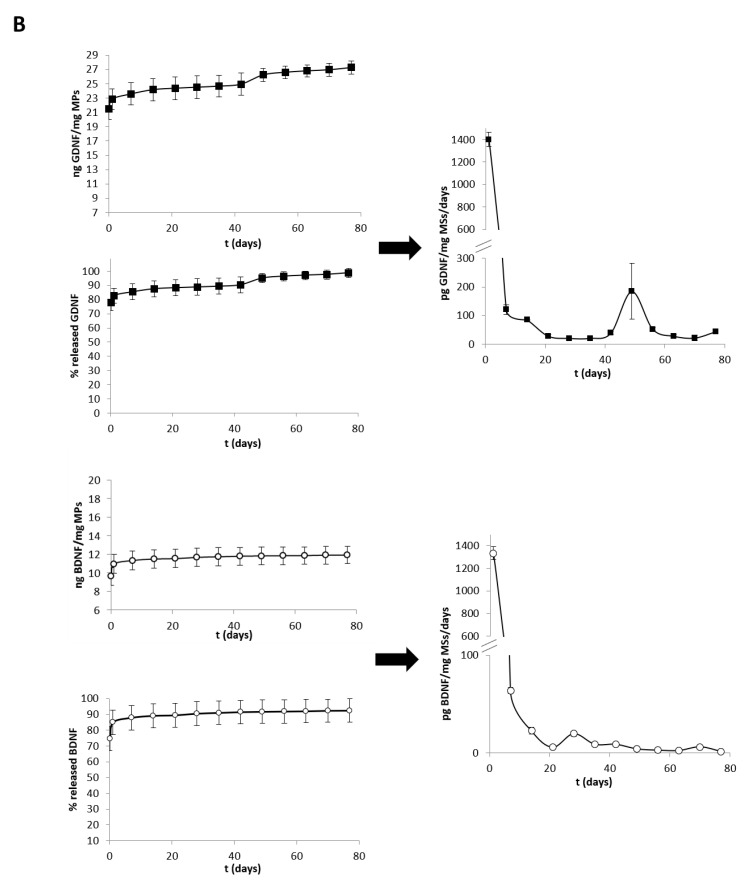
Release profiles of NTFs from PLGA/VitE matrices are presented expressed as cumulative ng NTF/mg MSs, cumulative % of total loaded protein and the release rate of NTF (pg NTF/mg MSs/day). (**A**) GDNF release from GDNF-loaded MSs. (**B**) GDNF (■) and BDNF (○) release from GDNF/BDNF-loaded MSs. Release media: PBS (pH 7.4) with 1.0% of BSA and 0.02% sodium azide.

According to Figure 2, the initial releases in the first 24 h (burst effects) were 11.64 ± 0.89 ng GDNF/mg MSs for MSs-GE20 (Figure 2A) and 22.86 ± 1.44 ng GDNF/mg MSs and 10.96 ± 1.02 ng BDNF/mg MSs for MSs-GBE40 (Figure 2B), corresponding to 61.02 ± 4.56% of GDNF encapsulated in MSs-GE20 and 84.95 ± 4.39% of GDNF and 84.61 ± 2.49% of BDNF encapsulated in MSs-GBE40. Subsequently, the MSs formulations released the entrapped proteins in a sustained fashion until the end of the study (day 77).

### 2.3. GDNF and GDNF/BDNF MSs are Safe for ARPE-19 and RF/6A Cells

**Figure 3:**
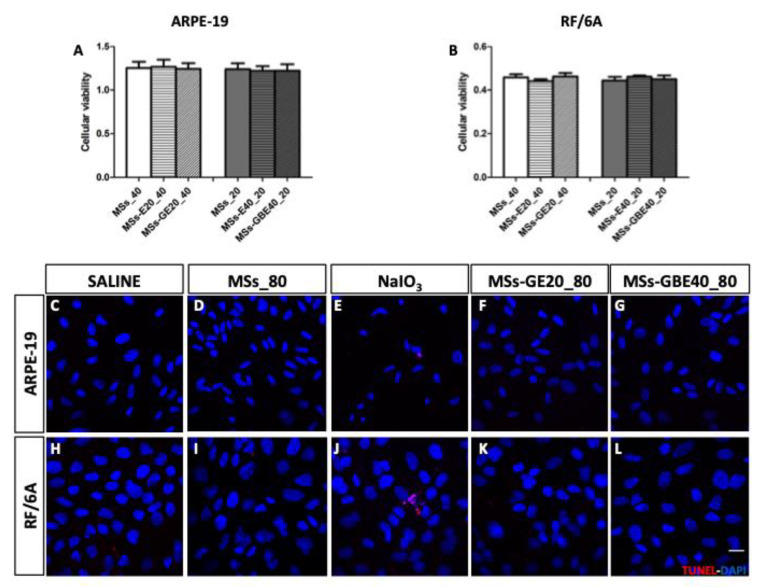
Safety and apoptosis of the delivery of neurotrophins by microspheres. MSs-GE and MSs-GBE showed no alterations in ARPE-19 (**A**) and RF/6A (**B**) cell viability measured by MTT. Blank PLGA MSs and PLGA/Vit E MSs (MSs-E20 and MSs-E40) controls also showed similar cell viability values in ARPE-19 (**A**) and RF/6A cells (**B**). Apoptosis was detected by Terminal deoxynucleotidyl transferase (TdT) dUTP Nick-End Labeling TUNEL (red) and nuclei were stained with DAPI (blue). TUNEL positive cells were only found in ARPE-19 (**E**) and RF/6A (**J**) cells after NaIO_3_ treatment for 24 h (1500 µg/mL for ARPE-19 cells and 500 µg/mL for RF/6A). TUNEL signal was absent in ARPE-19 (**C**,**D**,**F**,**G**) and RF/6A (**H**,**I**,**K**,**L**) cells after treatment with MSs_80, MSs-GE20_80 and MSs-GBE40_80. Blank MSs (MSs_80); GDNF/VitE(20)-loaded PLGA MSs (MSs-GE20_80); GDNF/BDNF/VitE(40)-loaded PLGA microspheres (MSs-GBE40_80). Scale bar 20 µm. *n* = 4 for MTT assay and *n* = 3 for TUNEL detection.

Neither MSs-GE20 nor MSs-GBE40 groups showed any sign of toxicity with regards to MTT measurements (expressed in absorbances) in ARPE-19 and RF/6A cells compared to the saline group after 24 h (Figure 3A,B, respectively). Moreover, blank PLGA MSs and PLGA/Vit E MSs (MSs-E20 and MSs-E40) controls did not show cytotoxicity neither in ARPE-19 cells nor in RF/6A cells (Figure 3A,B, respectively).

Additionally, TUNEL-staining is commonly used to detect DNA fragmentation, which is a hallmark of late cell apoptosis. NaIO_3_ induced retinal degeneration in in vivo studies, therefore it was used a positive control [55]. TUNEL assay was performed to determine whether GDNF/BDNF-loaded MSs induced cytotoxicity or contributed to apoptotic death in ARPE-19 and RF/6A. TUNEL assay demonstrated that GDNF and GDNF/BDNF-loaded MSs did not induce late apoptosis to ARPE-19 (Figure 3F,G) and RF/6A cells (Figure 3K,L), even at higher doses than those used for functional (migration and angiogenesis) and cell viability (MTT) studies. DAPI staining revealed no alterations of cellular morphology after treatments in ARPE-19 (Figure 3C–G) and RF/6A (Figure 3H–L) cells. Similarly, ARPE-19 (Figure 3C) and RF/6A (Figure 3G) cells incubated with blank PLGA MSs for 24 h did not show apoptotic cells. Apoptotic cells were only detected in NaIO_3_ (positive control) treated ARPE-19 and RF/6A cells (Figure 3E,J, respectively, and Figure S2).

### 2.4. Wound Closure Analysis: Migration in ARPE-19 and Angiogenesis in RF/6A Cells

**Figure 4:**
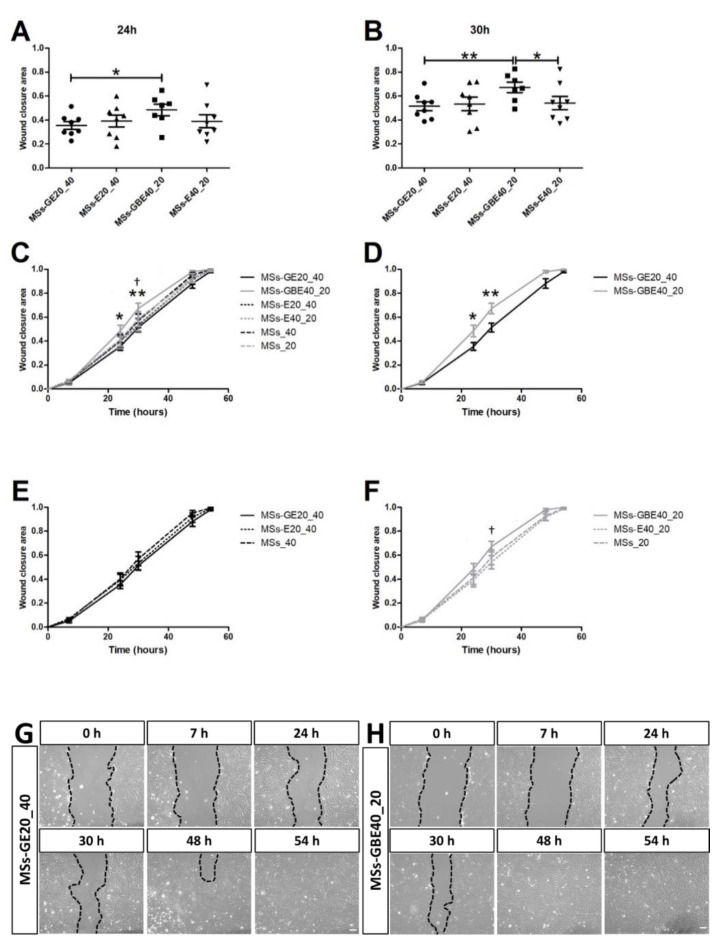
Wound closure area in ARPE-19 cells. MSs-GBE (−) treated cells showed a more closed wound area than MSs-GE (−) treated cells both at 24 h (**A**) and 30 h (**B**) from scratch (*p* <0.05 and *p* < 0.01, respectively) and than MSs-E20_40 (**- - -**) at 30 h (**B**, *p* < 0.05). Graphs (**C**–**F**) and representative images (**G**,**H**) show a different pattern in timeline migration between MSs-GBE and MSs-GE treated groups in ARPE-19 cells at 0, 7, 24, 30, 48 and 54 h after scratching. Black dotted lines indicate the wound borders at the different time points and treatments. Blank MSs (MSs_20) and (MSs_40); GDNF/VitE(20)-loaded PLGA MSs (MSs-GE20_40); GDNF/BDNF/VitE(40)-loaded PLGA microspheres (MSs-GBE40_20). Scale bar: 100 µm. *n* = 6–8. * *p* < 0.05 and ** *p* < 0.01 MSs-GBE vs. MSs-GE; † *p* < 0.05 MSs-GBE vs. MSs-E40_20.

The wound closure area in ARPE-19 cells from wound healing assay for MSs-GBE, MSs-GE and their respective controls is represented in Figure 4. Among all timepoints tested, there was no statistically significant difference in wound area recovered at 0, 7, 48 and 54 h (Figure 4G,H). In contrast, the area recovered was significantly higher in MSs-GBE samples compared to MSs-GE after 24 h (*p* < 0.05, Figure 4A) and after 30 h (*p* < 0.01, Figure 4B) from scratch. Moreover, MSs-GBE treatment showed significantly higher recovered area than its control MSs-E40_20 at 30 h (*p* < 0.05, Figure 4B,C,F), whereas MSs-GE treatment wound closure area remained similar to MSs-E20_40 at all time points (Figure 4B,C,E). A comparison of all time-lapses for all studied groups revealed differences in wound closure pattern. While MSs-GE closure pattern was similar to its control group (Figure 4E), MSs-GBE showed faster migration and therefore reaching total closure earlier than its control group (Figure 4F).

The evolution in migration between MSs-GBE and MSs-GE treated groups was different, as depicted in Figure 4D,G,H and Figure S3A,B. MS controls (MSs-E20, MSs-E40 and blank PLGA MSs) did not differ in migration pattern (Figure S3C–F). Representative images also show the abovementioned differences in wound closure area for MSs-GBE and MSs-GE treated groups (Figure 4G,H).

**Figure 5:**
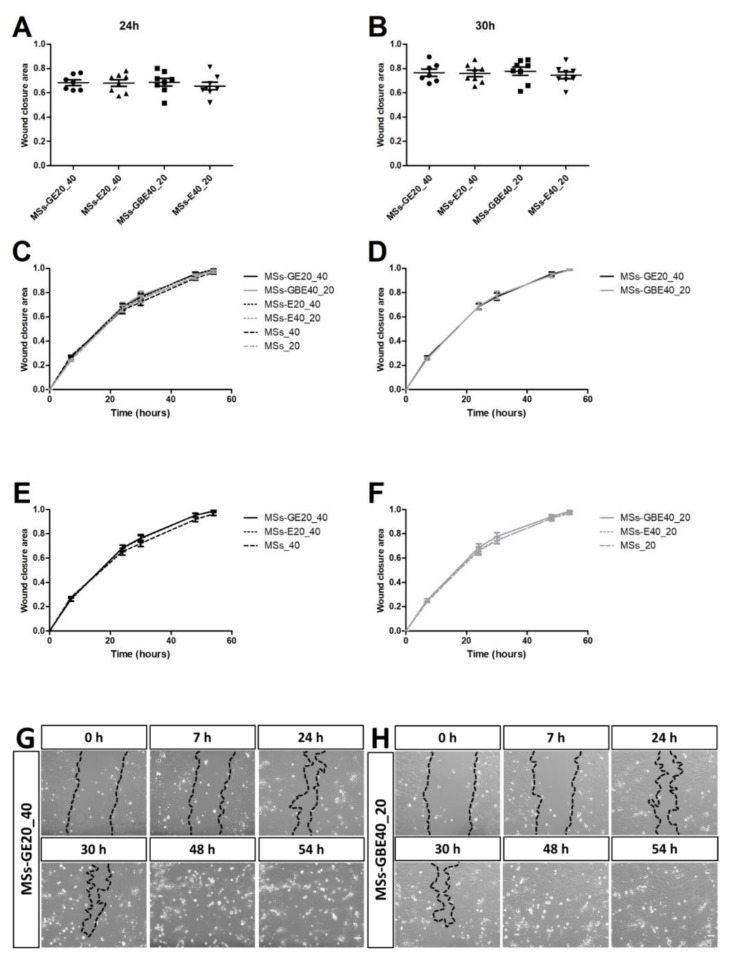
Wound closure in RF/6A cells represented by scatter plot and representative images. No statistically significant differences were found at 24 and 30 h (**A**,**B**) post-scratching. Moreover, wound closure pattern were similar for both treatments, MSs-GE (−) and MSs-GBE (−) in RF/6A cells at 0, 7, 24, 30, 48 and 54 h after scratch as shown in graphs (**C**–**F**) and representative images (**G**,**H**). Black dotted lines indicate the wound borders at the different time points and treatments. Black dotted lines indicate the wound borders at the different time points and treatments. Blank MSs (MSs_20) and (MSs_40); GDNF/VitE(20)-loaded PLGA MSs (MSs-GE20_40); GDNF/BDNF/VitE(40)-loaded PLGA microspheres (MSs-GBE40_20). Scale bar: 100 µm. *n* = 7–9.

In contrast to the observations in ARPE-19 cells, wound closure area and pattern was similar for all groups of study in RF/6A cells, and all statistically significant results between them at any time point was found (Figure 5A–F and Figure S4A–F). Representative images also showed a similar wound closure area for MSs-GBE and MSs-GE treated groups (Figure 5G,H).

### 2.5. Multi-Loaded MSs Injection Preserves Retinal Structure and RGCs Survival In Vivo

**Figure 6:**
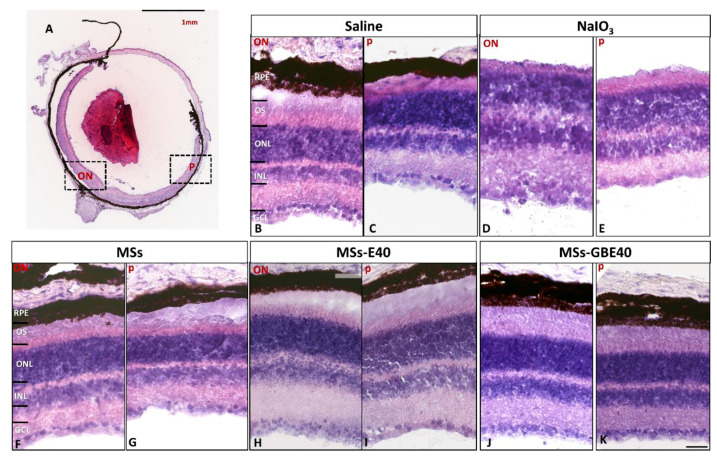
Histology (hematoxylin and eosin staining) of retinas one week after intravitreal injection. (**A**) Whole eye section showing an optic nerve (ON) and peripheral (p) framed areas observed. Retinal section from eye injected with saline (**B**,**C**), sodium iodate (**D**,**E**), MSs (**F**,**G**), MSs-E40 (**H**,**I**), MSs-GBE40 (**J**,**K**). No alterations (swelling, vacuoles, missed cells) were observed in any studied group. Scale bar: 1 mm (**A**) and 100 µm (**B**–**I**). Blank MSs (MSs); MSs/VitaminE(40) (MSs-E40), GDNF/BDNF/VitE(40)-loaded PLGA microspheres (MSs-GBE40). Abbreviations: RPE: retinal pigment epithelium, OS: outer segments, ONL: outer nuclear layer, INL: inner nuclear layer, GCL: ganglion cell layer, ON: optic nerve, p: periphery.

Taking into account the efficacy observed in the multi-loaded (GDNF/BDNF) system to increase RPE migration in vitro, we investigated its effect on retinal structure, apoptosis of retinal cells, and RGC survival in vivo in mice after one week from injection. Using hematoxylin-eosin staining, we confirmed the safety of MSs-GDNF/BDNF intravitreally injected (Figure 6). Retinal layers showed similar thickness in all groups observed vs. saline injection. The results showed that the eyeball tissue structure was normal and we did not find alterations (swelling, vacuoles, missed cells) either in optic nerve (ON) areas nor in peripheral areas in eyes injected with MSs (D,E), MSs-E20 (F,G), MSs-E40 (H,I), MSs-GE20 (J,K), MSs-GBE40 (L,M).

**Figure 7:**
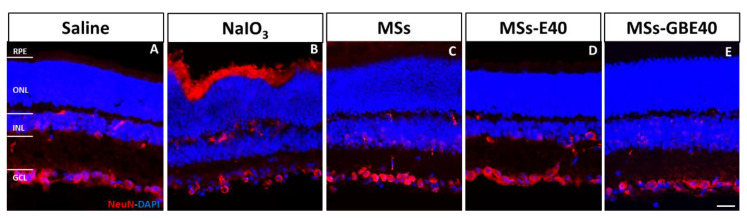
Immunofluorescent staining with anti-NeuN in mice retinas. NeuN (red) labeling is observed in a few cells in INL and mainly in GCL. Images show no alterations in GCL after intravitreal injection of saline (**A**), MSs (**C**), MSs-E40 (**D**) and MSs-GEB40 (**E**). Alterations in GCL were only found in sodium iodate injected animals (**B**). Nuclei of retinal cells were stained with DAPI (blue). Blank MSs (MSs); MSs/VitaminE(40) (MSs-E40), GDNF/BDNF/VitE(40)-loaded PLGA microspheres (MSs-GBE40). Scale bar: 20 µm. Abbreviations: RPE: retinal pigment epithelium, ONL: outer nuclear layer, INL: inner nuclear layer, GCL: ganglion cell layer.

NeuN is a DNA-binding protein that identifies most mature neuronal populations and as marker for RGCs. Subsequently, immunofluorescence of anti-NeuN in cross retinal sections was used to identify possible alterations in GCL (Figure 7). Fluorescence imaging showed NeuN positive cells in INL and GCL without alteration after injection of MSs (Figure 7B), MSs-E40 (Figure 7C), MSs-GBE40 (Figure 7D) vs. Saline (Figure 7A). The eyes injected with NaIO_3_ showed a decrease in NeuN positive cells (Figure 7B).

### 2.6. Absence of Apoptotic Cells after MSs Injection

**Figure 8:**
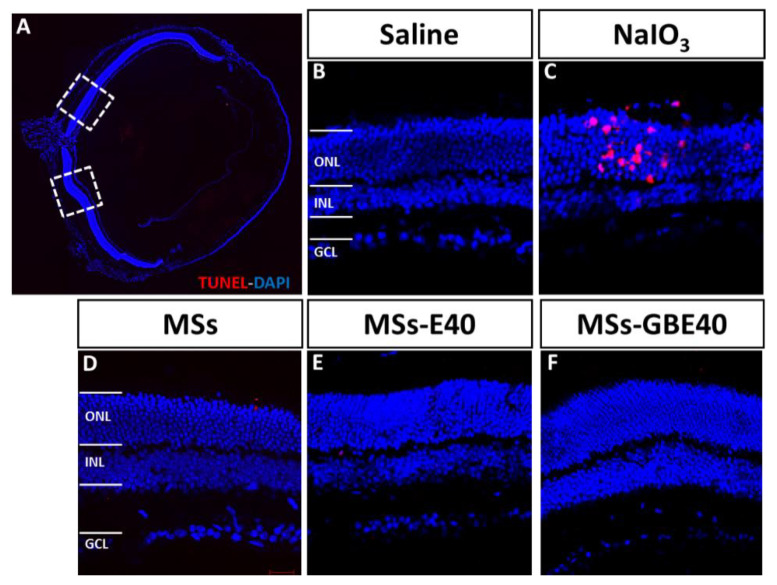
TUNEL staining of retinal tissue. Representative micrographs of retina sections were evaluated for apoptosis by TUNEL assay at 1 week after intravitreal injection. (**A**) Whole eye section shows the retinal areas observed. (**B**) Retinal section from eye injected with saline without TUNEL positive cells. (**C**) Retinal section from eye injected with sodium iodate, a control positive of apoptosis. TUNEL-positive cells were identified with red fluorescence retinal section from eyes injected with MSs, MSs-E40 and MSs-GBE40 (**D**,**E**,**F**, respectively). TUNEL-positive cells were not found in eyes injected with PLGA and MSs. Nuclei of retinal cells were stained with DAPI (blue). Blank MSs (MSs); MSs/VitaminE(40) (MSs-E40), GDNF/BDNF/VitE(40)-loaded PLGA microspheres (MSs-GBE40). Abbreviations: ONL: outer nuclear layer, INL: inner nuclear layer, GCL: ganglion cell layer. Scale bar: 20 µm.

By using a fluorescein-conjugated dUTP TUNEL-staining, we investigated the direct cytotoxic effects of intravitreal injection of MSs on a cellular level. We observed TUNEL-positive cells in the retina after NaIO_3_ administration in the photoreceptor layer, and a loss of retinal cells in INL and GCL (Figure 8C). However, in saline (Figure 8A), MSs (Figure 8D), MSs-E40 (Figure 8E), MSs-GBE40 (Figure 8F) injected eyes we did not find apoptotic cells in retinal samples.

### 2.7. Long-Term RGC Survival in Culture: Bioactivity of NTFs Released from the Microspheres

**Figure 9:**
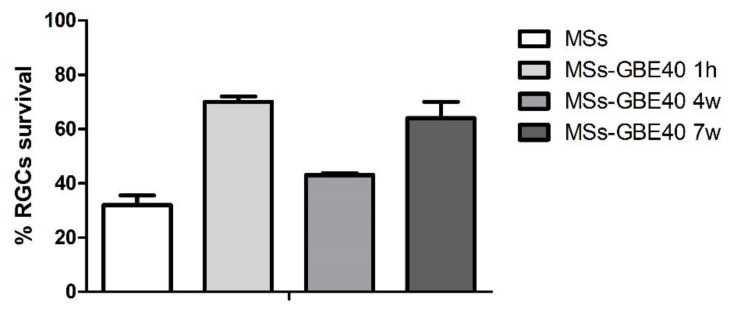
Bioactivity of GDNF/BDNF was demonstrated at 1 h, 4, and 7 weeks of the release study, increasing RGC survival by 70%, 43%, and 64%, respectively, compared to Blank MSs. Blank MSs (MSs); GDNF/BDNF/VitE(40)-loaded PLGA microspheres (MSs-GBE40).

In vivo safety of the multi-loaded NTFs observed at short-term evaluation was complemented with survival analysis of RGCs by TUNEL assay in isolated RGC cultures 24–48 h post-plating. Figure 9 illustrates that RGC survival increased by 70%, 43%, and 64% after exposition of release media, containing the neurotrophins, obtained at time points of 1 h, 4 weeks and 7 weeks of the MSs-GBE40 release study, respectively. On the contrary, in the absence of MSs-GBE40 (Saline), only 32% of RGCs survived.

## 3. Discussion

**Scheme 1:**
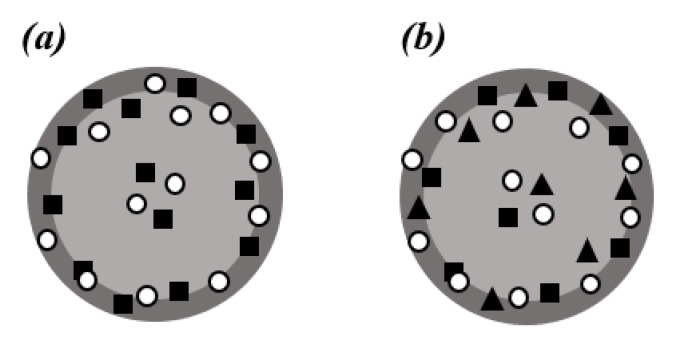
MSs formulation hypothesis (■ = GDNF protein; ○ = fatty additive; ▲ = BDNF protein). (**a**) GDNF/VitE(20)-loaded PLGA microspheres (MSs-GE20); (**b**) GDNF/BDNF/VitE(40)-loaded PLGA microspheres (MSs-GBE40).

The treatment of ocular multifactorial diseases with a combination of active substances is currently in the research pipeline [48]. In fact, the combination of two or more active substances has already demonstrated positive effects by blocking different pathways of the diseases at multiple levels. From a technological point of view, the microencapsulation of NTFs jeopardizes their biological activity because of the ease of rapid degradation and instability of the proteins [56,57,58]. Consequently, it is extremely important to use an appropriate method to encapsulate these biotechnological products to ensure the maintenance of bioactivity during and after the microencapsulation process. In a previous study from one of our groups, we reported a new microencapsulation method based on the solid-in oil-in water (S/O/W) emulsion solvent evaporation technique aimed to ensure the integrity of the biotechnological products [59]. This strategy involved the use of an oily additive, vitamin E, to incorporate GDNF in its solid form in PLGA-MSs promoting biological stability. Moreover, the incorporation of vitamin E provided additional protection because of its antioxidant properties [60]. That formulation promoted the survival of RGC and their axons for at least 11 weeks in a rat glaucoma model. The stability of the microencapsulated and then released protein was demonstrated by Western blot and bioassays studies in retinal cells [59,61]. The same technological strategy was successfully used in the present work, where we included not one protein, but two biotechnological products in their solid state in the inner organic phase of the emulsion. In addition, the MSs developed in the present experimental work were optimized to a small enough size that allowed their intravitreal injection as an aqueous suspension through standard needles, and, at the same time achieved a high enough concentration to control the release of the active compound(s). The porosity observed in the microspheres surface was previously explained as a consequence of the presence of an oily additive (VitE) in the inner phase of the emulsion during the microencapsulation procedure. According to previous works, the initial rapid diffusion of the organic solvent to the external phase of the emulsion during microencapsulation process might create a hardened shell retaining vitamin and protein. The existence of VitE on this concentrated layer close to the surface might promote the slow diffusion of the remaining organic solvent, forming the small pores observed on the MSs surface (Scheme 1) [59]. The GDNF and BDNF release profiles from the PLGA/VitE MSs were characterized by a high initial burst, previously explained by the already mentioned predominant disposition of the loaded proteins in the proximity of MSs surface. After the initial burst, the remaining protein(s) were sustained and delivered in the order of pg of protein per mg of microsphere per day for 77 days. Such small amounts of NTFs have been demonstrated to be neuroprotective in the retina [59]. This preliminary prototype allowed us to evaluate whether the co-microencapsulation and co-delivery of both NTFs could be an interesting strategy to treat retinal diseases with ongoing RPE and RGCs damage. Further technological approaches will be used in future to increase the protein loading capacity of the microsystem based on PLGA/VitE matrices and, simultaneously, reduce the initial release observed.

After the MSs were characterized and the NTFs release was measured, their safety was assessed on epithelial and endothelial cells. Our results showed that the MSs had a safe profile, even at higher doses than those used in migration and angiogenesis assays. The in vitro toxicity assay revealed that the presence of MSs and release of NTFs did not affect the viability of ARPE-19 and RF/6A cells compared to the control, and no alterations in cell morphology and retinal layers were observed in the mice. Additionally, in contrast to what was observed in sodium iodate-treated cells, no apoptosis of ARPE-19 and RF/6A cells and mouse retinal cell was observed following treatment with the MSs and release of GDNF and BDNF, which was similar to non-charged MSs and saline-treated cells.

After the in vitro safety confirmation, the main results of our work demonstrated that the combination of GDNF and BDNF was efficient in accelerating RPE cell migration. In contrast, GDNF alone was did not significantly increase RPE cell migration [62]. In agreement with our results, intravitreal administration of GDNF has shown beneficial effects in repairing RGCs [23] and restoring photoreceptors [26,27]. Additionally, BDNF has been observed to be downregulated in oxidative stress-related environment (high-glucose) in ARPE-19 cells [28]. In our in vitro study, only the combination of both NTFs showed a beneficial effect at the doses used. In humans, proliferating peripheral RPE cells have demonstrated the ability to migrate to central senescent regions of the retina to recover age-related central RPE loss [29]. Interestingly, mature RPE is held in a state of quiescence by the adjacent neural retina, and RPE cells proliferate in response to retinal degeneration [30]. Our results, although preliminary, suggest that the combination of GDNF/ BDNF could facilitate the acceleration of this response. This is in line with the results of Wang et al. [61], which demonstrated that the use of a collagen tube combined with both GDNF and BDNF improved functional regeneration of laryngeal nerves more than the use of a collagen tube with a single NTF did [62]. Furthermore, it has been demonstrated that BDNF promotes cell differentiation and inhibits inflammation under pathological conditions in the RPE [32,33], which agrees with our finding that RPE migration was improved by the co-release of BDNF/GDNF in PLGA/VitE MSs. Therefore, the artificial supplementation of these compounds might offer an interesting therapeutic benefit.

VitE has antioxidant effects and could be an effective adjuvant in the treatment of retinal diseases [63,64]. In fact, clinical trials have already demonstrated that oral antioxidant therapy reduced the risk of AMD development in 25% of patients treated [65,66]. However, the presence of this additive in the microcarriers proposed in this work did not provide any short-term pharmacological benefits; our results comparing MSs and MSs-E did not show any difference. This result agrees with those reported by Sakamoto et al. [67].

Following its effectiveness in increasing RPE cell migration, we demonstrated the in vivo safety of the multi-loaded MSs by validating our approach for further in vivo evaluation. This was with the aim of repairing RPE damage in experimental models, such as that induced by intravenous sodium iodate [68]. In our work, no apoptotic cells were found in the retinal structure and no morphological retinal alterations were observed one week after the multi-loaded MSs intravitreal injection. In addition, RGCs were observed to be conserved in vivo one week after the multi-loaded MSs intravitreal injection. The data obtained also suggest that GDNF and BDNF co-encapsulated and co-released from PLGA-VitE MSs maintained their bioactivity (and, hence, the three-dimensional integrity) following MSs elaboration and degradation during the release study.

Previous studies have already reported the possibility of using NTFs as anti-angiogenic agents [69,70]. For example, pigment epithelium-derived factor (PEDF) was demonstrated to inhibit the migration of endothelial cells in a dose-dependent manner [71] and reduce pathological vessel formation (CNV) in the eye using a sustained non-viral PEDF release system [72,73]. Therefore, the possibility of using NTFs as anti-angiogenic agents is an interesting platform for the treatment of vascular retinal pathologies. However, in our wound healing assay, the released GDNF alone or combined with BDNF from the PLGA/VitE MSs was not able to reduce the choroidal endothelial cell migration. Surprisingly, GDNF alone and in combination with BDNF did not show a beneficial effect on RF/6A choroidal endothelial cells. This could be explained by the specific response of endothelial cells to VEGF increase and anti-VEGF treatments. Probably, the appropriate therapeutic strategy for endothelial cells would be a combination with anti-VEGF molecules. Currently, the first-line treatment is anti-VEGF therapy, but intravitreal injection of anti-VEGF at high concentrations during the chronic treatment could be less beneficial than constantly released low doses of anti-VEGFs. Our results did not demonstrate differences in the area of wound closure covered, and further studies are needed to test different doses/formulations and technical approaches such as tube formation on Matrigel or choroid explants. Nevertheless, the therapeutic approach described herein needs to be further analyzed in combination with anti-VEGF therapy.

RPE tears and intraocular inflammation have been reported after anti-VEGF intravitreal administration [74] and an increased progression of GA [20,75]. In addition, the progression of atrophy was significantly related to the number of injections received [16]. However, it is not clear whether these effects are a consequence of the natural course of the disease or are provoked by the anti-VEGF injection [20]. GA could be a complication of VEGF inhibition at the RPE, due to the natural course of AMD leading to RPE damage [20]. Indeed, the absence of soluble VEGF could lead to the development of focal choroidal atrophy and RPE loss in mice [16]. Conversely, in large, controlled trials of anti-VEGF drugs in diabetic macular oedema and retinal vein occlusion, macular atrophy attributable to these drugs has not been detected [76,77,78]. Furthermore, in AMD patients under anti-VEGF therapy, RPE atrophy spares the retinal regions beyond the arcades, which are also exposed to VEGF inhibition [68]. Despite the initial trigger, the consequence is that the RPE is damaged, and our combination of BDNF/GDNF in PLGA/VitE MSs could contribute to preventing or restoring that damage by improving RPE cell migration and promoting the survival of primary RGCs from mice, at least after seven weeks.

One of the limitations of the present study is that the observed RPE cell migration occurred during the initial burst release of proteins from the MSs [79]. While a high initial release is generally associated with protein delivery systems, slow and maintained protein release has demonstrated long-lasting therapeutic effects [59,80]. These findings are an important advancement that contributes to the knowledge of the beneficial role of RPE cell migration. Thus, additional studies should be designed to test the effect of long-term delivery of these formulations in vitro using seven-day released supernatants of the MSs, and also to confirm the protein stability even at longer periods of release time than the seven weeks performed in this paper. Therefore, the therapeutic relevance of the tested system for chronic diseases where RPE degeneration is present and sustained amounts of active substances are required should be further investigated. As an in vitro study, cells were not exposed to the functioning retinal environment. Therefore, one of the main limitations of this research is the use of an immortalized cell line in which some transcription alterations could have occurred. Thus, future studies may include the use of primary RPE cells and in vivo experiments. In this sense, hESC-RPE cells have been validated as a relevant in vitro model to study the mechanisms of RPE-associated diseases, including retinal degenerations, showing very similar functional properties as the native RPE [81]. However, ARPE-19 is a well-validated and recognized cell line to study the RPE phenotype and function, exhibiting epithelial cell morphology and expressing several RPE specific genes, such as RPE65, among others. These cells not only maintain the RPE phenotype, but they also perform many of the known in vivo RPE functions, including assimilation of photoreceptor outer segments by phagocytosis [82].

To the best of our knowledge, this is the first study demonstrating that combining GDNF and BDNF in a novel, safe slow-delivery MS system may contribute to the improvement of RPE cell migration and RGC survival, although no effect was observed on endothelial cells at the doses used. Importantly, we have also confirmed the safety of the proposed system in epithelial and endothelial cells in vitro and in vivo. These findings may have therapeutic relevance for chronic diseases in which RPE degeneration is present and sustained amounts of active substances are required. Furthermore, different amounts of microparticles can be injected in the vitreous (that is, different doses) according to the patient’s needs. Consequently, this therapeutic approach could be a useful long-term therapy as a more personalized strategy for chronic retinal pathologies such as AMD and glaucoma, where atrophy of the RPE develops and a degeneration of RGCs occurs, in contrast to intravitreal implants and eye drops, which are typically administered at fixed dose.

## 4. Materials and Methods

### 4.1. Microspheres Elaboration

GDNF/VitE(20)-loaded PLGA microspheres (MSs-GE20) and GDNF/BDNF/VitE(40)-loaded PLGA microspheres (MSs-GBE40) were prepared using the S/O/W emulsion-solvent evaporation technique previously reported [59,83]. The first formulation was performed suspending 20 µg of recombinant human GDNF (R&D Systems, Minneapolis, MN, USA) in 20 µL of VitE (Sigma-Aldrich, Schnelldorf, Germany). In the second, 36 µg of recombinant human GDNF and 20 µg of recombinant human BDNF (R&D Systems) were suspended in 40 µL of VitE. In both cases, the suspensions were carried out throughout gentle sonication at low temperature (Sonicator XL, Head Systems, Inc., Farmingdale, NY, USA) for 30 s in order to reduce the risk of protein alteration [84]. Once formed the suspension, 1 mL of PLGA (Poly-(D,L-lactide-co-glycolide), ratio 50:50 -Resomer^®^ 503-Boehringer Ingelheim, Pharma Co., Essen, Germany) solution in methylene chloride (20% *w*/*v*) were added. The organic phases were emulsified in both phases with 5 mL of polyvinyl alcohol (PVA, 72,000 g/mol; Merck KgaA, Darmstadt, Germany) in MilliQ^®^ water solution (2% *w*/*v*) in a homogenizer (Polytron^®^ RECO, Kinematica GmbH PT 3000, Luzern, Switzerland) at 5000 rpm for 1 min. Afterwards, the prepared emulsions were subsequently poured onto 100 mL of an aqueous PVA solution (0.1%). The systems were maintained under constant stirred for 3 h at room temperature, to allow MSs to harden. After maturation, the formed MSs were washed in distilled water to remove PVA and separated according to their particle size by filtration using sieves (mesh size: 53, 38 and 20 µm) and a nylon mesh (1 µm). Finally, MSs were rapidly frozen (methanol/ice mixture) and lyophilized. Freezing: −60 °C/1 h, primary drying: −40 °C for 12 h and secondary drying: 20 °C/4 h and resulting cakes stored at −20 °C under dry conditions until required.

VitE MSs were elaborated according to the same protocol described before, except that no NTF was included, and two batches containing 20 µL and 40 µL of VitE, respectively, were elaborated: VitE (20)-loaded PLGA MSs (MSs-E20) and VitE (40)-loaded PLGA MSs (MSs-E40). Additionally, blank PLGA MSs in the absence of cargo were also prepared (MSs) [83].

### 4.2. Microspheres Characterization

#### 4.2.1. Production Yield (PY)

The PY% was calculated as the percentage of MS weight divided by the total amount of active substances, vitamin E and PLGA initially used in the formulation process Equation (1):

(1) $$\mathrm{PY}\;\%=\frac{{\mathrm{Weight}\;\mathrm{of}\;\mathrm{MSs}\;(W}_{1})}{{\mathrm{Total}\;\mathrm{weight}\;\mathrm{of}\;\mathrm{active}\;\mathrm{substances},\;\mathrm{vitamin}\;E\;\mathrm{and}\;\mathrm{polymer}\;(W}_{2})}\;\times \;100$$

#### 4.2.2. Morphological Evaluation

The morphology of MSs was assessed by means of scanning electron microscopy (SEM; Jeol, JSM-6335F, Tokyo, Japan) after a gold sputter-coating [85].

#### 4.2.3. Mean Particle Size and Particle Size Distribution

The MS mean particle size and particle size distribution were measured by light scattering in a Microtrac^®^ S3500 Series Particle Size Analyzer (Montgomeryville, PA, USA) [86]. Samples were prepared by suspending the microspheres in distilled water. The average particle size was expressed as the volume mean diameter in µm ± SD for 3 measurements per batch.

#### 4.2.4. Encapsulation Efficiency of NTFs

The content of encapsulated proteins in PLGA MSs was determined by a liquid/liquid extraction method [61,83]. Briefly, 5 mg of MSs were placed in 0.7 mL of methylene chloride. Upon dissolution of the PLGA, 0.7 mL of the diluent reactive provided in the ELISA Kit (PBS 7.4 and 1% BSA) was added. After vortex mixing, the samples were centrifuged at 7880 *g* for 15 min and the aqueous phase was collected. The extraction procedure was carried out four times to remove all the encapsulated proteins. The GDNF and BDNF content in the aqueous medium was determined by Enzyme-linked immunosorbent assay (ELISA) technique. The assays were performed in duplicate. The number of proteins recovered from each formulation was divided by the total mass added in the formulations to calculate proteins loading efficiency.

#### 4.2.5. In Vitro Release Studies of NTFs

Replicates of 5 mg of microspheres of each formulation were suspended in 1.5 mL of phosphate buffer saline (pH 7.4 isotonized with NaCl) containing 1% BSA as protein carrier and 0.02% sodium azide [59] as antimicrobial preservative. “Low binding” Eppendorf^®^ vials were used in all cases. Samples were placed in a shaker with a constant agitation speed of 100 rpm (Clifton Shaking Bath NE5, Nikel Electro Ltd. Avon, Weston-super-Mare, UK) at 37 °C. At pre-set times (1 h, 24 h and once a week until the end of the assay) the microspheres suspensions were gently centrifuged (490× *g* for 5 min) and the supernatant was recovered and replaced by an equal volume of fresh medium to continue the release test. The released proteins in supernatants were quantified by ELISA (R&D Systems). If necessary, aliquots from supernatants were diluted with the diluent reactive provided in the ELISA kits to obtain a protein concentration in the suitable ranges (15–1000 pg/mL for GDNF and 24–1500 pg/mL for BDNF).

### 4.3. ARPE-19 and RF/6A Culture

Human retinal pigment epithelial cells, ARPE-19 (ATCC^®^ CRL-2302) and a primary primate endothelial choroidal cell line, RF/6A (ATCC^®^ CRL-1780) were used in this study. ARPE-19 cells were grown to confluence in a standard incubator at 37 °C under humidified 5% CO_2_ conditions in Dulbecco’s modified Eagle’s medium (DMEM; Sigma-Aldrich, St Louis, MO, USA) containing 10% fetal bovine serum (FBS; Sigma-Aldrich), 1% fungizone, and L-glutamine penicillin-streptomycin (Sigma-Aldrich). RF/6A cells were grown to confluence in a standard incubator at 37 °C under humidified 5% CO_2_ conditions in Eagle’s minimum essential medium (ATCC^®^, 30–2003) containing 10% FBS (Sigma-Aldrich) 1% fungizone, and L-glutamine penicillin-streptomycin (Sigma-Aldrich).

### 4.4. Treatments

Cells were incubated with treatments according Table 2 for 24 h. The amounts of protein-loaded MSs for toxicity and wound closure assays were calculated to be able to release the same amount of GDNF within the first 24 h of the in vitro release study (~0.46 ng). The corresponding non-loaded protein MSs were tested in the same quantity. Sodium iodate (NaIO_3_, Sigma-Aldrich)—1500 µg/mL for ARPE-19 cells and 500 µg/mL for RF/6A—was used as a positive control for apoptosis.

### 4.5. Retinal Pigment Epithelium and Endothelial Cell Characterization

To verify that ARPE-19 and RF/6A cells preserved their phenotype, RPE65 and isolectin staining was performed [72,73]. Briefly, 10,000 ARPE-19 and 90,000 RF/6A cells were seeded on a 10 mm dish (Menzel-Glaser, Waltham, Massachusetts). Methanol was used for cellular fixing. Afterward, cells were washed with 1× PBS and permeabilized with 3% TritonX100, 0.5% Tween20. PBS-1% FBS was used for blocking nonspecific unions. Cells were incubated with the primary anti-RPE65 antibody for ARPE-19 cells (1:100; AB78036, Abcam, Cambridge, MA USA) and isolectin (1:240; B-1205, Vector Laboratories) for RF/6A cells diluted in 1% PBS-BSA at 4 °C for 24 h and washed once more with 1× PBS and then incubated with the secondary fluorescent antibodies goat anti-mouse 488 (A11029, Carlsbad, Life technologies, CA, USA) for RPE65 antibody and streptoavidin Alexa Fluor 594 (S32356, Life Technologies) for isolectin marker during 1 h. Nuclei were labelled with DAPI (40, 6-diamidino-2-phenylindole, Sigma-Aldrich) and images were obtained using a confocal microscope (LSM800, Zeiss, Oberkochen, Germany) (Figure S1).

### 4.6. Cellular Viability Assay MTT

The 3-(4,5-dimethylthiazol-2-yl)-2,5-diphenyltetrazolium bromide (MTT) reduction assay was used to determine cellular viability [86]. Ten thousand ARPE-19 and RF/6A cells were grown onto 96-well plates in DMEM with 10% FBS until confluence. Then, cells were cultivated for 1 additional week in 1% FBS–DMEM. MSs were added to the culture medium over 24 h at doses specified above and cellular viability was analyzed using the CellTiter 96^®^ AQueous One Solution Cell Proliferation Assay (Promega, Madison, WI, USA), following the manufacturer’s instructions.

### 4.7. Cell Migration Assays on ARPE-19 Cells and Angiogenesis Assays on RF/6A Cells Assessed by Wound Healing

Wound healing assay was used to evaluate the effect of MSs in ARPE-19 cell migration and on RF/6A cells for angiogenesis assessment [87,88]. For these experiments, 150,000 cells were seeded onto 24-well culture plates until confluence. Then, a linear wound was created in the middle of each well using a 10-µL micropipette tip. After culture media replacement, MSs were added. Photographs of 2 areas on each well were obtained at 0, 7, 24, 30, 48 and 54 h for ARPE-19 and RF/6A cells. Photographs were taken using a phase contrast inverted microscope equipped with a digital camera (Zeiss, Oberkochen, Germany). Every set of images was analyzed using Fiji software (a distribution of ImageJ) V1.48q to quantify the area closed for each group.

### 4.8. Terminal Deoxynucleotidyl Transferase dUTP Nick End Labelling (TUNEL) Assay to Detect Cell Apoptosis

Apoptosis was performed in coverslips of ARPE-19, RF/6A, RGCs culture and mice retinal sections using in situ cell death detection kit with TMR Red according to the manufacturer instructions (Roche, West Sussex, UK) (Roche #12 156 792 910) [72,73,89].

Sections containing the optic nerve head were selected for TUNEL assay TMR Red according to protocols in the manufacturer’s instructions (Roche, West Sussex, UK). Nuclei were labelled with DAPI (40, 6-diamidino-2-phenylindole, Sigma-Aldrich) and images were photographed using a fluorescence and confocal microscope (Axio Imager M1 and LSM800, Zeiss, Oberkochen, Germany).

### 4.9. Animals

The current study was conducted in accordance with the Association for Research in Vision and Ophthalmology (ARVO) Resolution on the Use of Animals in Ophthalmic and Vision Research and approved by the Ethics Committee for Animal Research of the University of Navarra (protocol approval number 094/19). Animals were housed and maintained at the Laboratory Animal Unit of the University of Navarra. Adult C57BL/6J mice (Charles River Laboratories, Wilmington, MA, USA) were used for RGC culture and adult wt. mice (C57BL/6J) male and female, aged six weeks (*n* = 6), obtained from Envigo Corporation (Indianapolis, IN, USA), were used for in vivo safety studies. All animals were housed and bred in a normal experimental room and exposed to a 12 h light/dark cycle with free access to food and water.

### 4.10. Intravitreal Injection and Microspheres Administration

Animals were initially anaesthetized with the gas anesthesia isofluorane (Merial; Animal Health Ltd., Essex, UK). Then, ketamine (75 mg/kg; Imalgene 1000; Merial Laboratories, Barcelona, Spain) and xylazine (10 mg/kg; Xilagesic 2%; Calier Laboratories, Barcelona, Spain) were used for subsequent anesthesia. Eyes were dilated with a mixture (1:4) of phenylephrine (7.8 mg/mL; Alcon Cusí, Barcelona, Spain) and tropicamide (3 mg/mL; Alcon Cusí) eye drops. Intravitreal injections (1 μL) of MSs were performed in mice using an automated pump-based perfusion system (LEGATO^®^ 130, KD Scientific Inc. Holliston, MA, USA). A 32 G needle coupled to a 10 µL syringe (Hamilton Bonaduz AG, Bonaduz, Switzerland) was inserted 1 mm posterior to the limbus with a 45° injection angle into the vitreous. Mice were divided into five groups: (1) saline (0.9% NaCl), (2) sodium iodate (100 mg/mL NaIO_3_), (3) MSs (8%), (4) MSs-E40 (8%), (5) MSs-GBE40 (8%).

### 4.11. Mice Retinal Sections

After 1 week, mice were sacrificed and enucleated eyes were fixed in 4% paraformaldehyde diluted in phosphate buffer (PB) (1 h, 4 °C). Following washing in PBS, the eyeballs were immersed in 30% sucrose solution (24 h, 4 °C) to ensure cryoprotection. Optimal cutting temperature compound (OCT) (Tissue-Tek-Sakura, Leiden, The Netherlands) was used for tissue embedding and stored at −20 °C. Finally, 12–14 μm cross sections were obtained using a cryostat (Microm HM550; Thermo Fisher Scientific) and frozen at −80 °C for subsequent use.

### 4.12. Conventional Histology and Neuronal-Specific Nuclear Protein (NeuN) Immunofluorescence

Conventional hematoxylin–eosin (H&E) staining for morphological observation of the retinal layers was performed in eye sections. In order to observe RGCs, three retinal sections containing the optic nerve head were selected for NeuN staining. Then, the sections were blocked with PBS, 1%; Triton X-100, 0.5%; sodium azide, 0.2% and BSA, 1% for 1 h at RT. Anti-mouse NeuN (1:500; Millipore, MAB377) was used for overnight incubation (4 °C). After washing with PBS, a donkey anti-mouse Alexa Fluor 596 (1:250; A10036; Life Technologies) antibody was added for 1 h (RT). Nuclei were counterstained with DAPI (Sigma-Aldrich). Then, sections were mounted with PBS-glycerol (1:1) and a fluorescence microscope (Axio Imager M1 and LSM800; Zeiss) was used for imaging and further analysis.

### 4.13. Isolation of RGCs and NTFs Bioactivity Asays

RGCs were isolated as previously described [90]. To isolate RGCs from mice, a magnetic-bead separation method was performed, using an antibody against a RGC-specific marker, Thy1.2, as the primary antibody. Rabbit anti-Thy1.2 conjugated with micrometal beads (CD90) was purchased from Multinyi Biotech (Auburn, CA, USA). After 30–60-day-old adult B6 mice were sacrificed, the retinas were dissected in Mg^2+^/Ca^2+^-free Hanks’ balanced salt solution (HBSS) and dissociated (incubating in HBSS, 1% papain, 5 U/mL DNase at 37 °C). Retinal cells were then transferred to a solution with the papain inhibitor 1% ovomucoid and triturated. Dissociated cells were treated with rabbit anti-Thy1.2 antibody conjugated to the micrometal beads in elution buffer (15 m, 4 °C). The cell suspensions were loaded onto a metal column and separated with the elution buffer in the presence and absence of a magnetic field. Selected cells were released from the magnetic column and rinsed and plated in conditioned media released from either blank MSs or MSs-GBE40 (release media containing NTFs released from the particles at 1 h, 4 weeks and 7 weeks). Approximately 1 × 10^5^ purified RGCs were seeded onto each well using twenty-four-well plates. The cultures were incubated at 37 °C in humidified 5% CO_2_ and 95% air. At 24–48 h post-plating, cultures were fixed and analyzed (TUNEL labeling, see Section 4.8) to determine the percentage of cell death in each condition.

### 4.14. Statistical Analysis

MTT and bioactivity results were analyzed by one-way ANOVA and wound healing results were analyzed by two-way ANOVA followed by post-hoc Bonferroni’s or Tukey’s correction. A *p*-value < 0.05 was considered to be statistically significant. Graphics and statistical analysis were performed using GraphPad software 5.0 (SPSS Inc., Chicago, IL, USA). Data are reported as mean ± standard error of the mean (S.E.M.).

## 5. Conclusions

In summary, we have provided a preliminary report of the novel beneficial role of a safe BDNF and GDNF sustained delivery system for RPE cell migration improvement. Therefore, our approach should be further investigated for the potential to serve as a useful therapy in a more personalized strategy for chronic retinal pathologies such as GA in AMD, where atrophy of the RPE or glaucoma develops. The use of microparticulate delivery systems might offer the additional advantage of controlling the amount of microparticles and, hence, the dose of the active compound(s) could be easily modified depending on the patient’s specific needs. Thus, this approach is congruent with future personalized therapies for individual patients. Moreover, it is becoming clear that reducing the injection frequency over time is one of the main factors contributing to a gradual loss of patients’ vision in the real-world scenario. Thus, the development of new therapeutic approaches that prolong the drug action by allowing sustained delivery of the active substance, thereby reducing the treatment burden while maintaining efficacy, remains as one of the greatest unmet needs of neurodegenerative chronic ocular diseases [91]. In addition, it is the focus of new strategies combining anti-VEGF therapies for CNV with other molecules to improve neuroprotection and the quality and duration of responses, while reducing frequency of treatments and diminishing side effects [92].

## Figures and Tables

**Table 1:** MSs characterization in terms of production yield, mean particle size and neurotrophic factor (NTF) loading and encapsulation efficiency expressed as ng of protein per mg of MSs and as percentage of the initial number of proteins for microspheres preparations, respectively. Blank MSs (MSs); MSs/VitaminE(20) (MSs-E20); MSs/VitaminE(40) (MSs-E40); GDNF/VitE(20)-loaded PLGA MSs (MSs-GE20); GDNF/BDNF/VitE(40)-loaded PLGA microspheres (MSs-GBE40).

Formulation	Production Yield (%)	Size (µm)	Encapsulation Efficiency (EE) and Protein Loading (ng/mg)			
			GDNF/MSs ng/mg	BDNF/MSs ng/mg	EE GDNF (%)	EE BDNF (%)
MSs-GE20	83.7	24.7 ± 0.3	19.2 ± 2.9	-	21.0 ± 3.2	-
MSs-GBE40	84.1	23.9 ± 0.2	27.6 ± 0.7	12.9 ± 0.8	17.8 ± 0.2	15.41 ± 1.0
MSs	88.4	27.4 ± 0.8	-	-	-	-
MSs-E20	88.1	25.5 ± 0.6	-	-	-	-
MSs-E40	83.5	23.9 ± 1.4	-	-	-	-

**Table 2:** Microsphere (MS) formulations and amount of tested MSs in each assay.

Formulation	GDNF (µg)	BDNF (µg)	Vit E(µL)	Toxicity Assay		Wound Closure Assay		Apoptosis Assay		Safety In Vivo		RGCs Survival	
				MSs (µg)	Naming	MSs (µg)	Naming	MSs (µg)	Naming	MSs (µg)	Naming	MSs (µg)	Naming
MSs-GE20	20	-	20	40	MSs-GE20_40	40	MSs-GE20_40	40	MSs-GE20_40			-	-
MSs-GBE40	36	20	40	20	MSs-GBE40_20	20	MSs-GBE40_20	20	MSs-GBE40_20	80	MSs-GBE40_80	20	MSs-GBE40_20
MSs	-	-	-	20/40	MSs_20/MSs_40	20/40	MSs_20/MSs_40	20	MSs_20	80	MSs_80	20	MSs_20
MSs-E20	-	-	20	20/40	MSs-E20_20/MSs-E20_40	40	MSs-E20_40	20	MSs-E20_40	-	-	-	-
MSs-E40	-	-	40	20/40	MSs-E40_20/MSs-E40_40	20	MSs-E40_20	20	MSs-E40_20	80	MSs-E40_80	-	-

## Data Availability

All data are available within the manuscript and upon request to corresponding authors.

## Supplementary Materials

The following are available online at https://www.mdpi.com/1424-8247/14/1/50/s1. Figure S1: ARPE-19 and RF/6A cells phenotype in culture. Figure S2: TUNEL assay in ARPE-19 and RF/6A. Figure S3: Wound healing assay in ARPE-19 cells. Figure S4: Wound healing assay in RF/6A cells.
