# Supplementary material for: A Safe GDNF and GDNF/BDNF Controlled Delivery System Improves Migration in Human Retinal Pigment Epithelial Cells and Survival in Retinal Ganglion Cells: Potential Usefulness in Degenerative Retinal Pathologies
Source: Pharmaceuticals (Basel). 2021 Jan 11;14(1):50. doi: 10.3390/ph14010050 (PMC7827036; doi:10.3390/ph14010050)
Supplement: Supplementary file 1 [file pharmaceuticals-14-00050-s001.pdf]

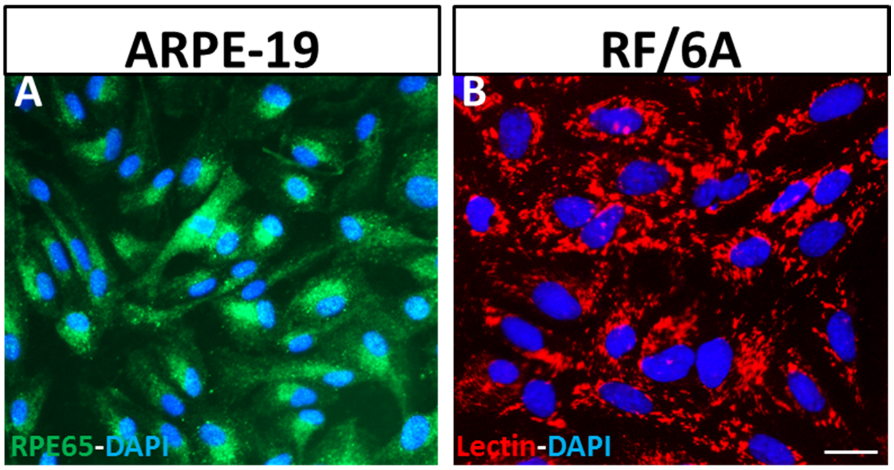

Figure S1. **ARPE-19 and RF/6A cells phenotype in culture.** (A) ARPE-19 cells were positive for RPE65 (green). (B) RF/6A cells were positive for lectin (red). Nuclei were stained with DAPI (blue). Scale bar: 20 μm.

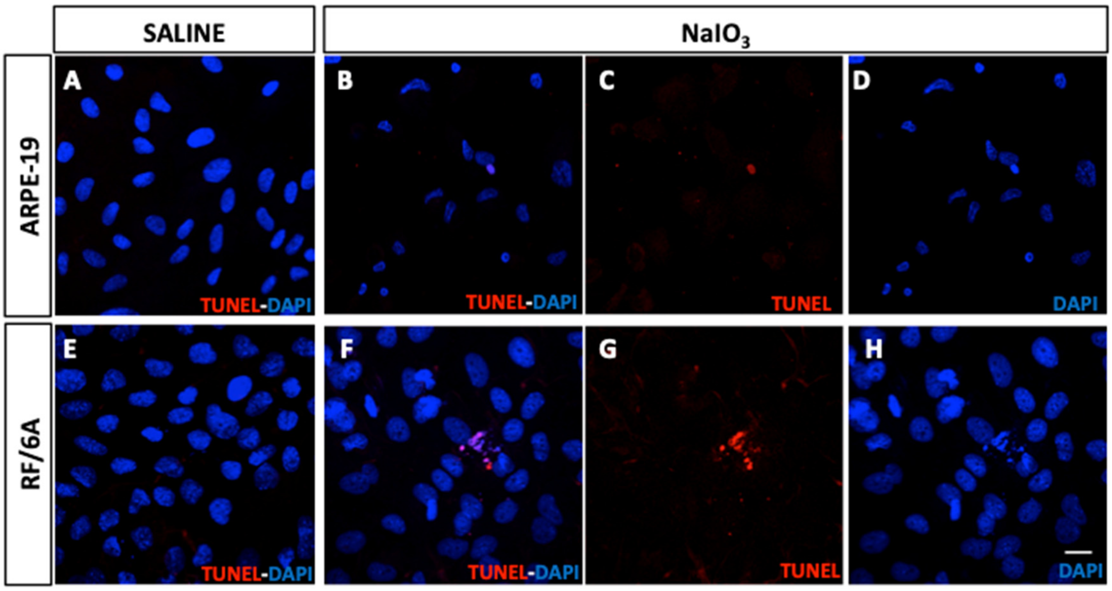

Figure S2. **TUNEL assay in ARPE-19 and RF/6A**. Saline (A and E) and sodium iodate (B-D and F-H) treated ARPE-19 (A-D) and RF/6A cells (E-H). Unmerged images are shown for sodium iodate treated samples to identify TUNEL signal (red, C and G) and DAPI (D and H).Scale bar: 20 μm

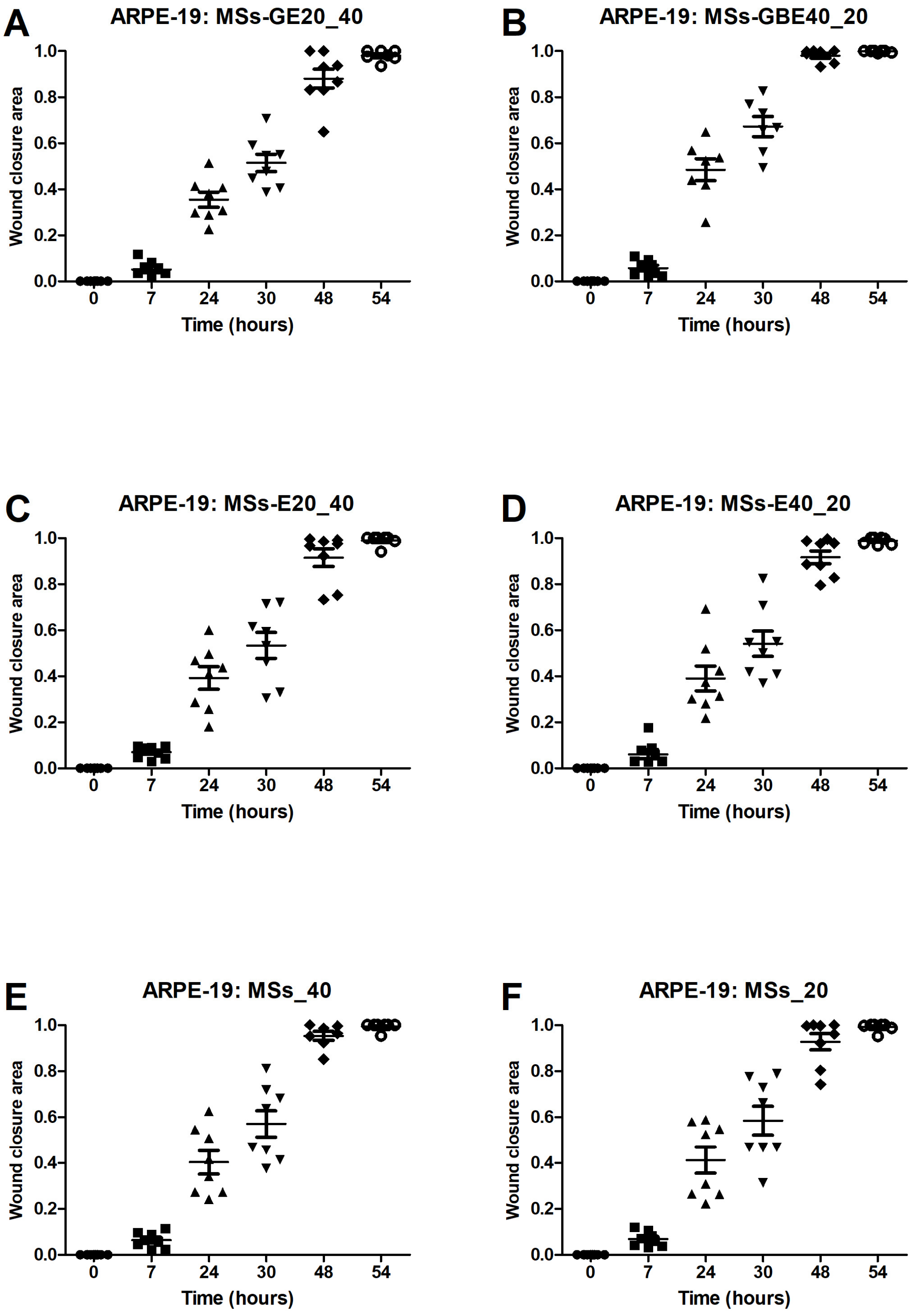

Figure S3. **Wound healing assay in ARPE-19 cells**. Scatter plot representation of wound closure area pattern in ARPE-19 cells (n=6-8) for the different study groups.

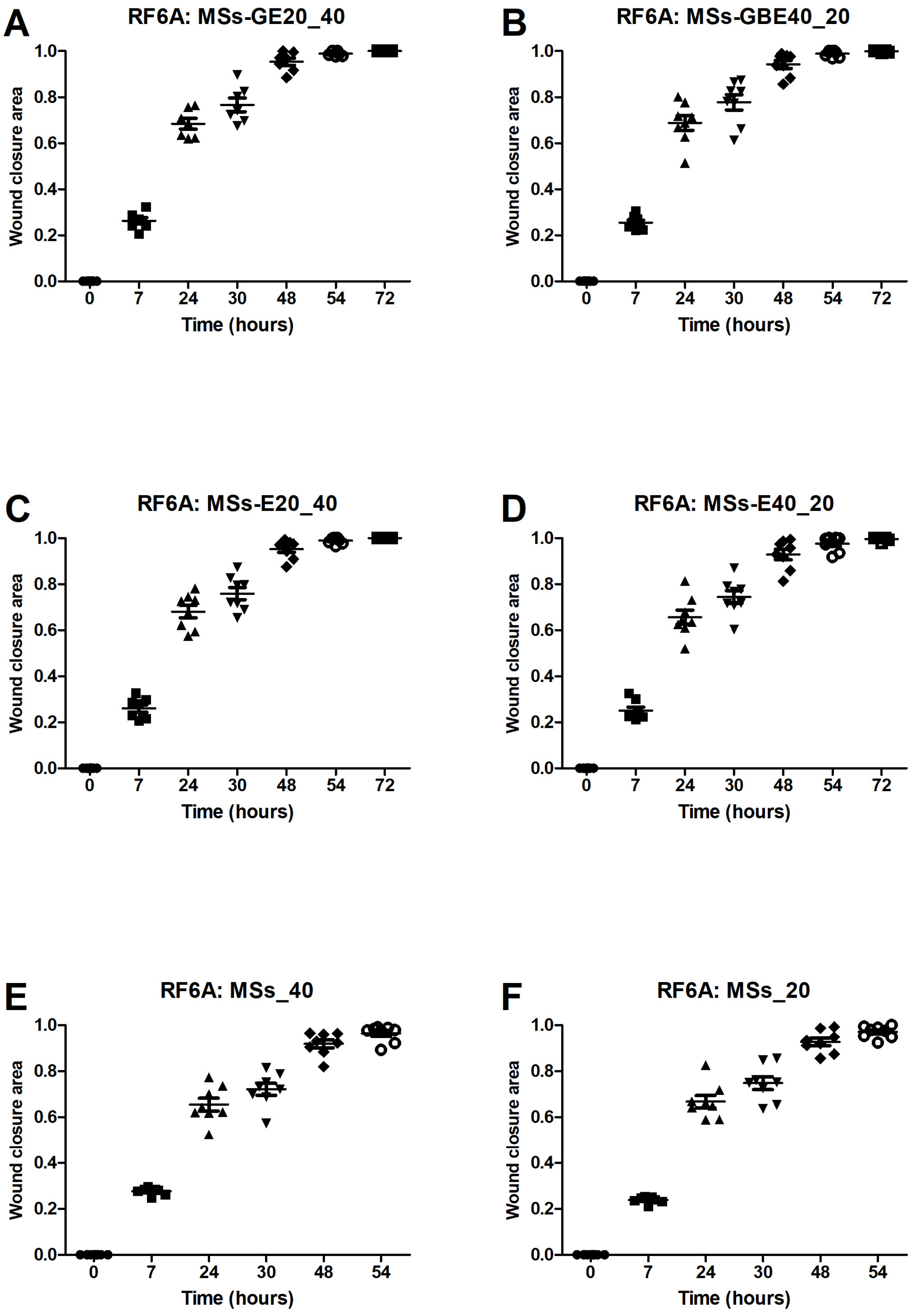

Figure S4. **Wound healing assay in RF/6A cells**. Scatter plot representation of wound closure area pattern in RF/6A cells (n=7-8) for the different groups of study.
